# Immunohistochemical profiling of the heat shock response in obese non-diabetic subjects revealed impaired expression of heat shock proteins in the adipose tissue

**DOI:** 10.1186/1476-511X-13-106

**Published:** 2014-07-01

**Authors:** Ali Tiss, Abdelkrim Khadir, Jehad Abubaker, Mohamed Abu-Farha, Irina Al-Khairi, Preethi Cherian, Jeena John, Sina Kavalakatt, Samia Warsame, Fahad Al-Ghimlas, Naser Elkum, Kazem Behbehani, Said Dermime, Mohammed Dehbi

**Affiliations:** 1 Department of Biomedical Research, Dasman Diabetes Institute, Kuwait City, Kuwait; 2 Fitness and Rehabilitation Center, Dasman Diabetes Institute, Kuwait City, Kuwait; 3 Department Biostatistics and Epidemiology, Dasman Diabetes Institute, Kuwait City, Kuwait; 4 Department of Biomedical Research, King Fahad Specialist Hospital, Dammam, Kingdom of Saudi Arabia; 5 Diabetes Research Centre, Qatar Biomedical Research Institute, Box: 5825, Doha, Qatar

**Keywords:** Heat shock protein, HSP, Exercise, ER stress, Obesity, Adipose tissue

## Abstract

**Background:**

Obesity is characterized by a chronic low-grade inflammation and altered stress responses in key metabolic tissues. Impairment of heat shock response (HSR) has been already linked to diabetes and insulin resistance as reflected by decrease in heat shock proteins (HSPs) expression. However, the status of HSR in non-diabetic human obese has not yet been elucidated. The aim of the current study was to investigate whether obesity triggers a change in the HSR pattern and the impact of physical exercise on this pattern at protein and mRNA levels.

**Methods:**

Two groups of adult non-diabetic human subjects consisting of lean and obese (n = 47 for each group) were enrolled in this study. The expression pattern of HSP-27, DNAJB3/HSP-40, HSP-60, HSC-70, HSP72, HSP-90 and GRP-94 in the adipose tissue was primarily investigated by immunohistochemistry and then complemented by western blot and qRT-PCR in Peripheral blood mononuclear cells (PBMCs). HSPs expression levels were correlated with various physical, clinical and biochemical parameters. We have also explored the effect of a 3-month moderate physical exercise on the HSPs expression pattern in obese subjects.

**Results:**

Obese subjects displayed increased expression of HSP-60, HSC-70, HSP-72, HSP-90 and GRP-94 and lower expression of DNAJB3/HSP-40 (P < 0.05). No differential expression was observed for HSP-27 between the two groups. Higher levels of HSP-72 and GRP-94 proteins correlated positively with the indices of obesity (body mass index and percent body fat) and circulating levels of IFN-gamma-inducible protein 10 (IP-10) and RANTES chemokines. This expression pattern was concomitant with increased inflammatory response in the adipose tissue as monitored by increased levels of Interleukin-6 (IL-6), Tumor necrosis factor-α (TNF-α), and RANTES (P < 0.05). Physical exercise reduced the expression of various HSPs in obese to normal levels observed in lean subjects with a parallel decrease in the endogenous levels of IL-6, TNF-α, and RANTES.

**Conclusion:**

Taken together, these data indicate that obesity triggers differential regulation of various components of the HSR in non-diabetic subjects and a 3-month physical moderate exercise was sufficient to restore the normal expression of HSPs in the adipose tissue with concomitant attenuation in the inflammatory response.

## Introduction

Obesity has become a major medical, social and economic burden worldwide by reducing both life quality and expectancy [1,2]. It also represents a major risk factor for various health disorders including insulin resistance, diabetes, hypertension and cardiovascular diseases (CVD). As physical inactivity, sedentary lifestyle and increased energy intake are key contributing factors to obesity, healthy diet and regular exercise are usually prescribed as a first line non-medical therapy to control obesity-related complications [3].

Obesity is characterized by a chronic, low-grade inflammation and an impaired intracellular stress defence system in key metabolic tissues including muscles, adipose tissue and liver [4]. While obesity-induced inflammation depends on a set of classical inflammatory mediators, the stress response is more complex and includes an impairment of the heat shock response (HSR) [5,6], excessive activation of the oxidative stress along with down-regulation of the cellular antioxidant defense system [7], dysfunction of the mitochondria [8], and the endoplasmic reticulum (ER)-mediated stress [4].

HSR is one of the important cellular endogenous components that allow the body to cope with stressful conditions including metabolic stress [9,10]. This response involves a set of highly conserved proteins called Heat shock proteins (HSPs) known also as molecular chaperones, glucose regulated proteins (GRPs) and other proteins essential for protection and recovery from tissue damage [11-13].

Intracellular HSPs execute their tasks by binding non-covalently to misfolded, aggregated and nascent proteins and either assist in their proper folding, solubility and their appropriate translocation or alternatively, eliminate them through the degradation pathway [13]. HSPs can also be released into the circulation and exert an immune-stimulatory effect by interacting with pattern recognition receptors, such as toll-like receptors, and thereby activate the host inflammatory response [14,15]. In mammalian cells, several families of HSPs with distinct functional classes have been described to date and they consist of HSP-110, HSP-90, HSP-70, HSP-60, HSP-40, HSP-27 and the small HSPs [16].

The best characterized heat shock protein is HSP-72, the inducible member of the HSP-70 family and its role in insulin resistance was recently a subject of intense investigations. Indeed, HSP-72, was shown to be associated with insulin resistance as its expression was reduced in type 2 diabetic patients [5,6] and this observations was confirmed in cellular and animal models [17,18]. Reduced expression of intracellular HSPs was also linked to CVD and metabolic syndrome, two chronic conditions that are complicated by obesity and diabetes [19,20]. Other studies reported attenuated cardio-protective role of HSPs with aging factor as this latter is well known to decrease the expression of HSPs [21,22]. By contrast to the protective role of intracellular HSPs, increased levels of circulating HSP-60 were founded in patients with acute myocardial infarction (AMI) and as such, HSP-60 was suggested as predictive marker of post-AMI adverse events [23,24].

The importance of the HSR against these chronic conditions was subsequently confirmed by using interventions that induce the HSR and showing a concomitant improvement of the clinical outcomes. These interventions include heat therapy [18,25] or electrical therapy [26], physical exercise [26], using pharmacological molecules that stimulate the heat shock response [10,27,28]. Based on these evidences, it was suggested that increasing HSPs expression could be considered a therapeutic strategy to protect from insulin resistance, diabetes and to reinforce vascular defense and delay or avoid clinical complications associated with CVD. While our knowledge on the mechanisms and the contribution of HSPs is well documented for other conditions, little is known about their dysregulation during the course of obesity, particularly in non-diabetic subjects.

In the current study, we investigated the expression levels of various HSPs in non-diabetic human adult obese and the impact of a 3-month moderate physical exercise on the expression of those proteins. Our results have shown that HSPs expression levels were increased in non-diabetic obese as compared to lean control subjects. Physical exercise, however, has normalized their levels in most cases. This suggests that, in non-diabetic obese, the HSR in human body is still coping with the obesity-related stress.

## Material and methods

### Study population

The study was conducted on adult non-diabetic male and female subjects consisting of 47 lean (20 ≤ BMI < 25 kg/m^2^) and 47 obese (30 ≤ BMI < 40 kg/m^2^). Informed written consent was obtained from all subjects before their participation in the study which was approved by the Review Board of Dasman Diabetes Institute (RA2010-003) and carried out in line with the guideline ethical declaration of Helsinki. Participants that followed any physical exercise within the last 6 months prior to this study, with prior major illness or intake of medications and/or supplements known to influence the body composition or bone mass were excluded from the study. White Blood Cell count (WBC) was performed for all enrolled subjects to ensure the absence of any recent inflammation due to pathogens or inflammatory diseases. The physical, clinical and biochemical characteristics of the participating subjects are shown in Table 1. The data on HSP-72 in obese subjects prompted us to add 10 diabetic obese subjects to the study to assess the expression levels of HSP-72 in obese with and without diabetes.

**Table 1:** Physical, clinical and biochemical characteristics of the subjects at baseline

	**Lean (n = 47)**	**Obese (n = 47)**	***P-value***
***Physical and Clinical characteristics***			
AGE (year)	38.0 ± 9.4	39.1 ± 8.3	*0.51*
Gender (M/F)	20/27	23/24	*0.44*
BMI (kg/m^2^)	22.60 ± 1.97	34.95 ± 2.93	*<0.0001*
Weight (kg)	62.02 ± 10.32	96.36 ± 14.71	*<0.0001*
Height (m)	1.65 ± 0.10	1.66 ± 0.11	*0.77*
PBF (%)	27.43 ± 5.23	39.29 ± 4.98	*<0.0001*
SLM (Kg)	42.03 ± 9.29	53.66 ± 11.09	*0.0002*
Waist (cm)	79.39 ± 15.59	108.61 ± 13.20	*<0.0001*
Hip (cm)	92.29 ± 14.67	118.55 ± 8.27	*<0.0001*
Resting HR (beat/min)	80.71 ± 14.51	77.43 ± 8.15	0.73
SBP (mmHg)	113.00 ± 10.81	127.50 ± 11.89	0.01
DBP (mmHg)	76.43 ± 6.33	82.00 ± 10.14	0.13
V_O2 Max_ (ml/kg/min)	21.63 ± 3.76	17.48 ± 4.83	0.03
***Metabolic markers***			
Cholesterol (mmol/l)	5.14 ± 0.90	5.26 ± 0.98	*0.22*
HDL (mmol/l)	1.47 ± 0.50	1.11 ± 0.25	*<0.0001*
LDL (mmol/l)	3.18 ± 0.88	3.41 ± 0.94	*0.09*
TG (mmol/l)	0.90 ± 0.43	1.57 ± 0.93	*<0.0001*
Glucose (mmol/l)	4.96 ± 0.53	5.40 ± 0.85	*0.013*
HBA1C (%)	5.47 ± 0.42	5.91 ± 1.11	*0.0006*
C-peptide (ng/ml)	2.59 ± 0.71	3.08 ± 1.35	*0.04*
Glucagon (ng/ml)	0.65 ± 0.11	0.71 ± 0.15	*0.05*
GLP-1 (ng/ml)	2.62 ± 0.83	2.68 ± 1.54	*0.96*
Insulin (ng/ml)	2.54 ± 1.06	4.51 ± 2.11	*0.003*
Leptin (ng/ml)	5.25 ± 2.95	8.41 ± 4.39	*0.0004*
PAI-1 (ng/ml)	3.19 ± 1.55	4.15 ± 1.53	*0.015*
***Inflammatory markers***			
TNF-α (pg/ml)	24.07 ± 8.30	28.94 ± 14.23	*0.96*
IL-1β (pg/ml)	1.20 ± 0.47	1.35 ± 0.85	*0.89*
IL-6 (pg/ml)	5.29 ± 2.02	4.97 ± 2.32	*0.37*
IL-10 (pg/ml)	1.97 ± 2.00	2.53 ± 2.84	*0.87*
IP-10 (pg/ml)	365 ± 139	556 ± 224	*0.0002*
MCP-1 (pg/ml)	9.06 ± 2.73	10.20 ± 4.41	*0.89*
MIP-1a (pg/ml)	6.93 ± 3.54	10.74 ± 6.08	*0.04*
RANTES (ng/ml)	1.32 ± 0.68	1.78 ± 0.77	*0.01*
***Oxidative stress markers***			
ROS (mM)	1.41 ± 0.28	1.51 ± 0.15	*0.35*
TBARS (μM)	1.18 ± 0.54	1.53 ± 0.49	*0.20*

Data are presented as mean ± SD. Non-parametric Mann–Whitney test was used to determine significance of difference in means between lean and obese groups.

### Exercise protocol and anthropometric measurements

All eligible subjects were enrolled to a supervised exercise program at the Fitness and Rehabilitation Center (FRC) of Dasman Diabetes Institute. Prior to exercise prescription, each individual underwent a symptom-limited maximal incremental cardiopulmonary exercise test “CPET” using an electromagnetically braked cycle ergometer (COSMED Quark, Italy). The CPET was primarily used to determine the maximum heart rate (max HR) as well as the response to aerobic exercise as measured by the maximum oxygen consumption (VO₂ Max) for each subject. The exercise training involves a combination of both moderate intensity of aerobic exercise and resistance training using either treadmill or cycling. Each exercise session includes 10 minutes warming-up and cooling down steps at 50-60% of max HR, along with 40 minutes of the prescribed exercise program at 65-80% of max HR. For the duration of the 3-month period, participants exercised 3 to 5 times per week and they were instructed to reach and maintain the recommended heart rate range. This was achieved by regular monitoring of the heart rate during the aerobic training. Strength training was performed 2 to 3 times a week according to the program plan. Exercise intensity, duration and blood pressures were recorded for each session. All trainings were supervised by qualified fitness professionals from FRC. To assess the effectiveness of the exercise, the same, previously mentioned, physical stress and fitness tests were performed for all subjects at the end of the exercise program. Anthropometric measurements were taken at the baseline and after 3 months of exercise. Whole-body composition including body fat percent was determined by dual-energy radiographic absorptiometry device (Lunar DPX, Lunar radiation, Madison, WI).

### Blood and tissue sampling

Venous peripheral blood and subcutaneous adipose tissue biopsies were obtained before (baseline) and after 3 months of exercise. PBMCs were prepared from blood using Ficoll-Hypaque density gradient centrifugation method. Plasma and serum were prepared using vacutainer tubes and then aliquoted and stored at −80°C. Subcutaneous superficial adipose tissue biopsies (~200 mg) were obtained from the periumbilical area by surgical biopsy after a local anesthesia. Once removed, the biopsy was rinsed in cold PBS, divided into 4 pieces and stored appropriately until assayed.

### Blood biochemistry, inflammatory and metabolic markers

Glucose, lipid profiles and HbA1c were measured in our clinical laboratory using standard clinical kits. Plasma levels of inflammatory and metabolic markers were measured with bead-based multiplex immunoassays and the Bio-Plex®-200 system (BioRad, Hercules, CA). This technology is based on the use of distinctly color-coded beads that can be conjugated with specific antibodies allowing simultaneous quantification of up to 100 different analytes in the same reaction. To measure the levels of inflammatory and metabolic markers, we used the human cytokine 27-plex panel kit and the human 12-plex diabetes kits, respectively (BioRad, Hercules, CA). A dual detection flow cytometer is used to sort out the different assays by bead colors in one channel and determine the analyte concentration by measuring the reporter dye fluorescence in another channel. Standard curves for each analyte were generated using the pre-mixed standards provided in the kits. The concentration of each analyte in each sample was determined from the appropriate standard curve using a 5 point-regression to transform mean fluorescence intensities into concentrations. Lipid peroxidation was assessed by measuring plasma levels of malonaldehyde, using TBARs Assay Kit (Cayman Chemical Company, Ann Arbor, MI). Serum levels of ROS were determined using the OxiSelect™ ROS Assay Kit (Cell Biolabs Inc, San Diego, CA). All the above assays were carried out according to the instructions of the manufacturers.

### Immunohistochemistry (IHC)

Formalin fixed, paraffin embedded adipose tissue samples were prepared and used to make sections for IHC studies as described previously [29]. Briefly, sections were deparaffinized and the antigens were retrieved at high-temperature using antigen unmasking solution (Dako, Glostrup, Denmark). The endogenous peroxidase was quenched using 3% H_2_O_2_ (Merck, Schuchardt, Gemany) for 30 min at room temperature (RT). Sections were blocked with 5% fat-free milk for 1 h at RT followed by 1% BSA for 1 h and then, incubated at 4°C for overnight with the corresponding primary antibodies. After washing, sections were stained with horseradish conjugated secondary antibody (Dako, Glostrup, Denmark) for 1 h at RT. Colors were developed using DAB kit (Dako, Glostrup, Denmark) and sections were counterstained with hematoxylin (Sigma Aldrich, St. Louis, MO). The primary antibodies used in IHC were raised against HSP-27, HSP-60, and HSP-72 (Enzo LifeSciences. Inc, Lausen, Switzerland), HSC-70, HSP-90, and GRP-94 (Cell Signaling Technology Inc., Danvers, MA), and DNAJB3/HSP-40 (Proteintech Group. Inc, Chicago, IL). Negative controls were performed by omitting the application of primary antibodies. Biopsies from breast cancer, kidney cancer, colon, testis and prostate were used as positive controls for IHC as recommended by the manufacturers.

Samples from lean, obese before and after exercise were stained and analyzed within the same batch. For each slide, a series of 3 images were generated from different areas of the tissue section under an objective magnification of 20x. The quantification of the IHC data was done using ImageScope software version 11.1 (Aperio, Vista, CA). The algorithm Positive Pixel Count v9 of this software provided the percentage of positive staining (number of stained pixels) as compared to the whole image area. Then, each annotated picture was manually checked against the original slide picture to ensure the correct matching between the positive staining and the software annotation. Finally, the staining analysis results of the 3 images obtained from each tissue section were averaged.

### Quantitative real time-PCR (qRT-PCR)

Total RNA was extracted from frozen PBMCs using AllPrep RNA/Protein Kit (Qiagen, Inc., Valencia, CA). The cDNA was synthesized from total RNA sample using High Capacity cDNA Reverse Transcription Kits (Applied Biosystems, Foster City, CA). qRT-PCR was performed on Rotor gene Q-100 system using SYBR Green normalized to GAPDH (Qiagen, Inc., Valencia, CA). Relative gene expression between the groups was assessed by using the ΔΔCT method [30] and GAPDH as internal control for normalization. The primers used in this study are summarized in Additional file 1: Table S1.

### Western blot analysis

Western blots were carried out on whole PBMC extracts prepared in RIPA buffer (50 mM Tris–HCl pH 7.5, 150 mM NaCl, 1 mM EDTA, 1% Triton ×100, 0.5% Sodium deoxycholate, 0.1% SDS and supplemented with a mini complete protease inhibitor cocktail (Roche Diagnostics, Laval, Quebec). Protein concentration was determined by Bradford method using globulin as a standard. For Western blot, 20 μg of proteins were resolved on 10% or 12% SDS-PAGE gels. Proteins were then transferred onto PVDF membranes, blocked with 5% non-fat dried milk in Tris-buffered saline containing 0.05% Tween 20 (TBST) for 1 h at RT and then probed with the primary antibody for overnight at 4°C. After washing, the membranes were incubated with horseradish peroxidase-conjugated secondary antibody for 2 h at RT. Finally, protein bands were visualized by chemiluminescence and the images were captured by using the Versadoc 5000 system (BioRad, Hercules, CA). Primary antibodies used here were the same mentioned in the IHC section. Actin (Santa Cruz Biotechnology, Santa Cruz, CA) was used as internal controls. For densitometric analysis, the intensity of the bands was determined using Quantity One Software (BioRad, Hercules, CA).

### Statistical analysis

Statistical analyses were performed with SAS version 9.2 (SAS Institute Inc, Cary, NC). Unless otherwise stated, all descriptive statistics for the variables in the study were reported as means ± standard error. Non-parametric Mann–Whitney test was used to determine significance of difference in means between the lean and obese groups before exercise. Paired t-test was used to determine significance of difference in means between the obese group before and after exercise. Correlations between variables were calculated with the Spearman’s rank correlation test. Differences were considered statistically significant at P-values less than 0.05.

## Results

### Baseline characteristics of the study population

**Figure 1:**
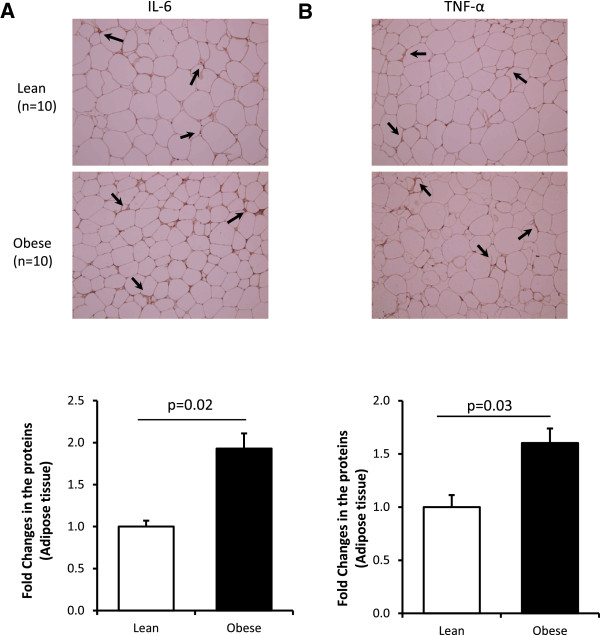
**Inflammation markers increase in the adipose tissue of obese subjects.** Analysis of IL-6 **(A)** and TNF-α **(B)** expression by IHC using the subcutaneous adipose tissue from lean (n = 10) and obese (n = 10) non-diabetic participants. Aperio software was used to quantify positive staining (indicated by arrows on the IHC slides) as detailed in Material and Methods section. The data are presented as fold changes in obese compared to lean subjects. Non-parametric Mann–Whitney test was used for statistical analysis.

In this study, we selected non diabetic subjects consisting of 47 obese (23 males and 24 females) and 47 age*-*matched lean (20 males and 27 females) from our cohort study. The physical, clinical and biochemical characteristics of the study population at baseline are summarized in Table 1. As expected, body mass index (BMI), percent body fat (PBF), waist and hip circumferences were all significantly higher in obese than in the control subjects (P < 0.0001). Obese subjects had also higher systolic blood pressure (P = 0.01) but lower maximal oxygen consumption (V_O2 Max_; P = 0.03). HDL levels were significantly lower in obese (P < 0.0001) whereas TG levels were higher in those subjects (P < 0.0001). Although the study population is not diabetic, obese subjects had higher glucose and HbA1c levels (P = 0.013 and P = 0.0006; respectively). Likewise, the levels of C-peptide, Glucagon and insulin were higher in obese subjects (P < 0.05). The levels of leptin and PAI-1 were also higher in obese subjects (P = 0.0004 and P = 0.015; respectively). Obese subjects had increased inflammatory response as monitored by the inflammatory chemokines IP-10, MIP-1a and RANTES (P = 0.0002, P = 0.04 and P = 0.01; respectively) but there was no significant difference in the plasma levels of the classic inflammatory markers IL-6 and TNF-α between lean and obese groups. However, the endogenous expression of IL-6 and TNF-α protein and mRNA in the adipose tissue were significantly increased in obese subjects (Figure 1). There was also no significant difference between the two groups in the levels of oxidative stress markers ROS and TBARS (Table 1).

### Obesity triggers differential expression pattern of components of the heat shock response

**Figure 2:**
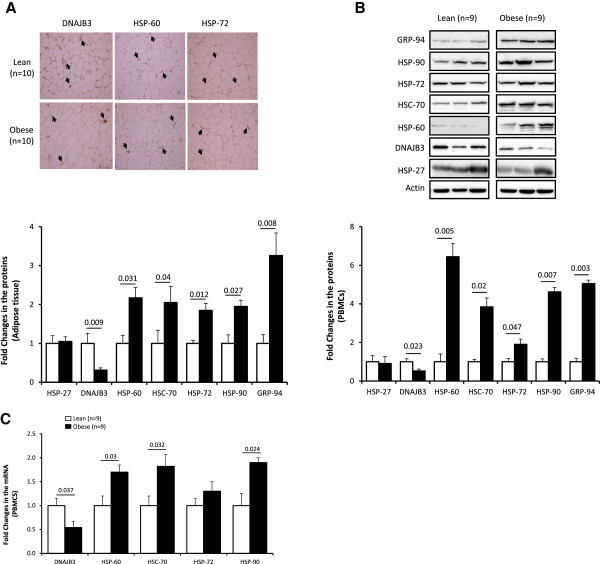
**Protein and mRNA Expression profiles of components of the HSR in non-diabetic obese subjects. (A)** Representative slides of HSPs and chaperone expression levels using IHC and the subcutaneous adipose tissue from lean and obese participants (n = 10 for each group). Aperio software was used to quantify positive staining from lean and obese participants as detailed in Material and Methods. **(B)** Representative Western blots of lean and obese subjects showing the expression pattern of various HSPs and chaperones. Total proteins were extracted from PBMCs of lean and obese participants (n = 9 for each group) and detected using the indicated antibodies. The blots shown are representative of 3 experiments with consistent results. Densitometric quantification of the Western blots data. The bands of interest were quantified as described in materials and methods and the relative intensities were determined after correction with Actin. **(C)** Quantitative analysis of HSPs mRNA in lean and obese subjects using PBMCs (n = 9 for each group). Total mRNA was isolated and subjected to analysis using qRT-PCR. The quantified data are presented as fold changes in obese compared to lean subjects. Non-parametric Mann–Whitney test was used for statistical analysis.

To investigate the expression levels of various components of the heat shock response between lean and obese groups we initially used the subcutaneous adipose tissue to determine the expression pattern of seven HSPs at the protein level by immunohistochemistry (IHC). As shown in Figure 2A, there was more than 1.5-fold increase in the expression of HSP-60, HSC-70 HSP-72, HSP-90 and GRP-94 in obese group compared to lean group (n = 10 for each group; P < 0.05). By contrast, the expression of DNAJB3 was significantly reduced in obese group (Figure 2A; P = 0.009) and this was consistent with our previous finding [31]. The levels of HSP-27 remained however unchanged between the two groups (Figure 2A). As the amount of adipose tissue biopsies were limited to prepare whole tissue lysate for Western blot to validate the IHC data, we prepared whole cell lysates from PBMCs of lean and obese subjects (n = 9 for each group) and used them for Western blot analyses. Figure 2B confirmed the findings of the IHC study, i.e., no change in the expression levels of HSP-27 between the two groups, a significant increase in the expression of HSP-60, HSC-70, HSP-72, HSP-90 and GRP-94 in obese group (P < 0.05) and a decrease in the levels of DNAJB3 in obese group (P = 0.023). These data have been further validated at the mRNA levels in PBMCs by qRT-PCR that showed a similar trend of increase and decrease in the expression of various HSPs (Figure 2C).

Defect in the heat shock response in diabetic conditions is an established fact both in humans and animals in particular for HSP-72 protein [5,6,18]. The unexpected increase in the expression of HSP-72 in obese subjects prompted us carry out a comparison in the expression levels of HSP-72 protein in 10 diabetic obese using the adipose tissue. As expected, HSP-72 protein expression levels were significantly attenuated in this diabetic group as compared to non-diabetic obese subjects (data not shown).

### Correlation analysis

**Figure 3:**
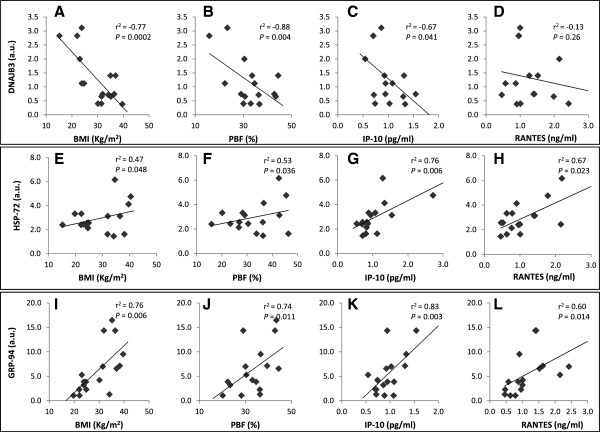
**Correlation analysis.** DNAJB3 **(A-D)**, HSP-72 **(E-H)** and GRP-94 **(I-L)** expression levels in adipose tissue, as quantified using IHC staining, were correlated with BMI, PBF, IP-10 and RANTES. Correlations were assessed using Spearman’s rank correlation coefficient.

In an attempt to understand the relationship between the levels of HSPs in the adipose tissue and various physical, clinical and biochemical parameters of our study population at baseline, we used Spearman’s rank test for correlation analysis. The most significant correlations were observed for DNAJB3, HSP-72 and GRP-94 as illustrated in Figure 3. Accordingly, there was a negative correlation between DNAJB3 levels with BMI, PBF and circulating levels of the inflammatory chemokine IP-10 (Figures 3A, B and C; P < 0.05). By contrast, both HSP-72 and GRP-94 levels correlated positively with BMI and PBF (Figure 3E, F, I and J, respectively; P < 0.05). Likewise, higher levels of HSP-72 and GRP-94 correlated positively with the circulating levels of both IP-10 and RANTES inflammatory chemokines (Figure 3G, H, K and L, respectively; P < 0.05). A similar but separated analysis of the mRNA levels of the HSPs confirmed most of those correlation trends obtained from relative protein expression levels using IHC (data not shown).

### Modulation of the heat shock response by physical exercise

**Figure 4:**
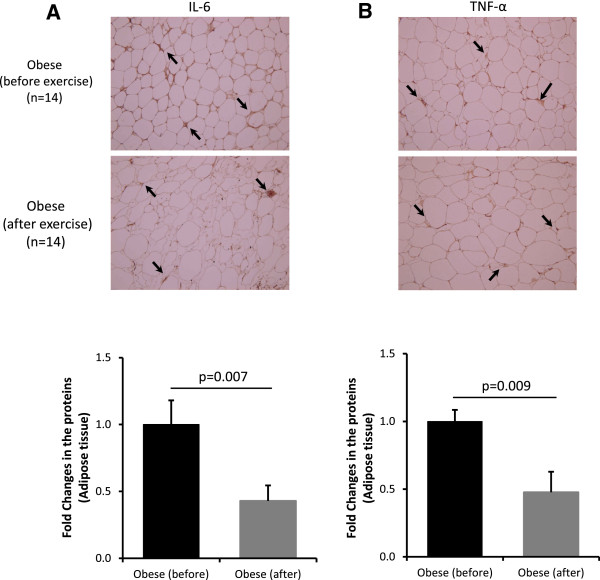
**Physical exercise restored IL-6 and TNF-α expression levels in obese subjects.** Analysis of IL-6 **(A)** and TNF-α **(B)** expression by IHC using subcutaneous adipose tissue from obese non-diabetic participants before and after exercise (n = 14 each). Aperio software was used to quantify positive staining (indicated by arrows on the IHC slides) as detailed in Material and Methods section. Quantified data are presented as fold of change. Paired t-test was used for statistical analysis.

**Figure 5:**
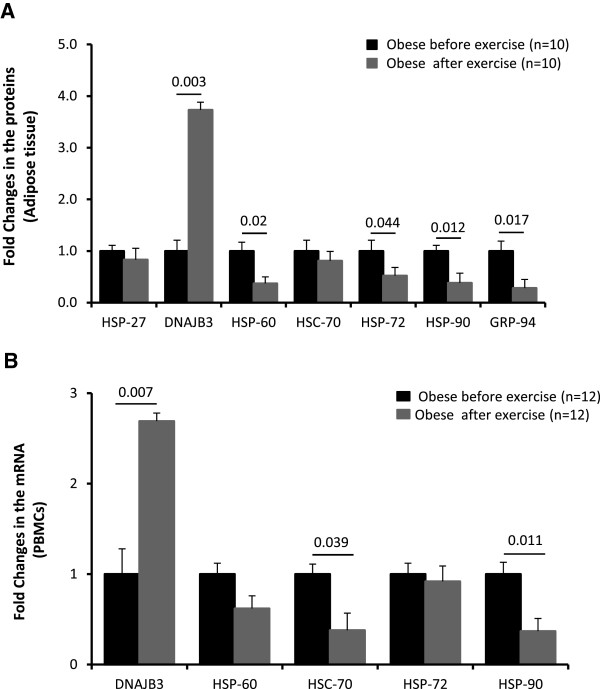
**Physical exercise restored HSPs expression levels in non-diabetic obese subjects. (A)** IHC staining using subcutaneous adipose tissue from obese before and after exercise (n = 10 each). Aperio software was used to quantify positive staining as detailed in Material and Methods. **(B)** qRT-PCR analysis of HSPs mRNA in PBMCs from obese subjects before and after exercise (n = 12 each). Quantified data are presented as fold of change. Paired t-test was used for statistical analysis.

Physical exercise is becoming the first line non-pharmacologic choice for the prevention and management for obesity and its complications. This prompted us to investigate the effects of physical exercise on the expression levels of HSPs. We previously reported that our 3-month physical exercise protocol that while it had no significant effect on improving the BMI of obese subjects, it reduced significantly the PBF and SBP an increased the maximal oxygen consumption as reflected by the V_O2 Max_[31]. Physical exercise was also associated with a reduction in the inflammatory response as indicated by reduced levels of IL-6, TNF-α and MCP-1 aside with clear decrease in TBARS and increase in ROS [31]. In the adipose tissue, the endogenous expression of IL-6 and TNF-α was significantly reduced in obese subjects by physical exercise (Figure 4A and B). The expression of RANTES and its CCR5 receptor were also attenuated by physical exercise in obese subjects [32].To investigate whether physical exercise had an impact on the expression of HSPs, adipose tissue collected from obese before and after the exercise program was analyzed by IHC. As shown in Figure 5A, our physical exercise protocol restored the normal expression of the HSPs tested to levels comparable to lean control group. Indeed, the levels of HSP-60, HSP-72, HSP-90 and GRP-94 proteins were all significantly decreased by physical exercise (P < 0.05), while the expression of DNAJB3 was significantly induced by physical exercise (Figure 5A). This effect of physical exercise on HSPs expression in obese subjects was also confirmed at the level of mRNA from PBMCs (Figure 5B).

## Discussion

Dysregulation of the heat shock response is a crucial event that leads to diabetes through the development of insulin resistance. However, the contribution of obesity to this dysregulation in humans has not yet been investigated. In the current study, we profiled the expression pattern of various components of the heat shock response in lean and obese subjects and established the possible correlations with various physical, clinical and biochemical parameters of the study population. We also investigated whether physical exercise could restore the expression of proteins showing differential expression between lean and obese subjects. The main findings of this investigation are: 1) obesity increased significantly the expression of the majority HSPs investigated while it had no effect on the expression of HSP-27 and reduced significantly the expression of DNAJB3-HSP-40, 2) the expression levels of HSP-72 in obese subjects are linked to the diabetes status of the study population, and 3) a 3-months physical exercise protocol was sufficient to restore the abnormal expression of HSPs with a concomitant decrease in the inflammatory response.

To the best of our knowledge, this is the first study that investigated simultaneous expression of components of the HSR in non-diabetic human obese subjects using adipose tissue as well as evaluating their response to physical exercise. It is worth noting that the observed expression pattern of the HSR between lean and obese subjects was not related to gender as both males and females have shown the same trend at mRNA and protein levels (data not shown).

The fact that we observed a similar pattern in the expression of the HSR in PBMCs and the adipose tissue indicate that HSR dysregulation is not only limited to the subcutaneous adipose tissue and thus PBMCs could be an interesting surrogate target for follow up studies to understand the mechanisms leading to dysregulated expression of the HSR by obesity.

It is well established that in obesity, the uncontrolled inflammatory reaction and impairment of the host defense system, plays an important role in the inhibition of insulin receptor signaling cascade and as a direct consequence, disruption of systemic metabolic homeostasis that leads to T2D [33]. Recently, it was proposed that T2D is the result of a metabolic paradigm in which metabolic inflammation; insulin resistance and impairment of the HSR work in a vicious cycle [34,35]. Our findings that investigated the sole contribution of obesity on the status of the heat shock response indicate that the defect in HSR observed previously in obesity-induced insulin resistance and diabetes [5,6,18] is more complex than what was initially thought and it presumably involves different mechanism of action.

The activation of the HSR is a crucial step required to maintain normal homeostasis in response to physiological and patho-physiological damages. It is well known that exposure to a preconditioning stress increases a cell's tolerance to subsequent stress, and this effect has been shown to be partially due to an increase in HSP synthesis; particularly HSP-27, HSP-60 and HSP-72 following the preconditioning stress [36]. Increased content of HSPs is thought to restore cellular homeostasis and remodelling, and protect against further cellular stress and damage in vulnerable tissue organs [37,38]. In the context of obesity, the observed dysregulation of the HSR can be suggested as a physiological response to alleviate metabolic stress in key tissue organs such as the adipose tissue and muscles. It is well established that obesity is associated with activation of JNK stress kinase [31,39] and enhanced inflammatory response as monitored by the endogenous levels of IL-6, TNF-α, RANTES and CCR5 receptor in the adipose tissue [32]. Thus, the up-regulation of the HSPs in obese subjects is presumably required to mitigate the inflammatory response and to alleviate various forms of metabolic stress response induced by obesity. It is not however impossible that the observed increase in the expression of HSPs in the current study could be a compensation for reduced expression of DNAJB3.

Several studies showed that interventions leading to induction of the HSR or chaperone therapy such as heat therapy [18,25], transgenic mice or liposomal delivery of HSPs [18], electrical therapy [26], physical exercise [40] and pharmacological drugs [27,28] were associated with beneficial outcomes as monitored by improved glucose homeostasis, enhanced insulin sensitivity, reduction of visceral adiposity and suppression of the chronic inflammatory state [16,41-43]. Nevertheless, it is worth noting that most of these studies investigated the status of the HSR in human and animal models of obesity-induced insulin resistance and diabetes and they mainly focused on the expression of HSP-72 and HSP-25/27 in skeletal muscles. In agreement with those studies, we found that HSP-72 expression levels in the subcutaneous adipose tissue were attenuated in a diabetic obese group as compared to non-diabetic obese subjects. In non-diabetics obese subjects, our data indicated that HSP-72 was increased in obese subjects as compared to lean subjects. The mechanisms underlying the differential regulation of HSP-72 in diabetics and non-diabetic obese subjects are still unknown. In line with this, previous studies have report discrepancy in the expression levels of HSP-72 depending on the tissues [44,45].

Another important aspect that was investigated in the current study is the effect of physical exercise on the expression pattern of components of HRS in obese subjects. Physical exercise is one of the highly recommended strategies to reduce weight and to improve the clinical outcomes in obese and diabetic patients although the exact molecular mechanisms underlying these beneficial effects are not yet well established. Our findings indicated that a 3-month aerobic exercise was sufficient to restore the expression of the HSR in obese subjects to normal levels observed in lean subjects. Under the same conditions, the endogenous levels of RANTES and its CCR5 receptor [32], IL-6 and TNF-α in the adipose tissue of obese subjects were significantly reduced by physical exercise (Figure 4). It is worth noting that the exercise affected similarly the expression pattern of the HSR in both male and female obese subjects (data not shown). Some previous studies have investigated the effect of a short time “acute” exercise on the expression of HSPs in males and females and the results were not consistent. For example, Gillum et al. [46] reported the effect of a single acute exercise reported that HSP-72 was more increased in men than women, whereas Njemini et al. [47] reported no difference in HSP expression between genders. Nevertheless, up to the best of our knowledge no report is available on a gender-linked effect of long-term exercise on the expression of HSP in human.

Our current findings are however contrasting previous studies that reported increased expression of HSPs by physical exercise [6,18,38,41,48]. One of the possible reasons for this discrepancy is that these investigations focused on acute effect of exercise on transient expression of HSPs either immediately after [6,18,41,48] or within 7 days of exercise using a single-session exercise [38]. The different behavior of HSPs from the adipose tissue in our study as compared to those previous studies could also be due to the non-damaging and long-term nature of our physical exercise protocol. Indeed, exercise is considered as a stress factor in particular when it is intensive and the type of exercise strongly influences the levels of circulating HSp-72 (eHSP-72) in blood for example [49]. For instance, in treadmill or downhill running aerobic exercise increased several times both cellular HSP-72 and blood eHSP-72 during and immediately after exercise [50,51]. In contrast, elbow flexion eccentric exercise doesn’t increase eHSP-72 [52]. The transient increase in HSPs could be assigned to the elevation of temperature in the tissues during exercise as temperature is critical for the activation of HSR in exercising mammals [53-55]. As the adipose tissue, in particular the subcutaneous one, is superficial and has less vascularisation, its temperature increase will be limited compared to other organs. For example the temperature of muscles and core body at the end of 45 min moderate treadmill exercise exceeded 39°C [38]. Thus, the observed elevated and long-lasting HSP levels in the cells may have better benefits, as compared to only transient effect, for the body to cope with the low-grade inflammation characterising obesity. Other authors have already suggested, in agreement with our current study, that HSP are expressed and induced by exercise in a tissue-specific manner [38,56].

In summary, we demonstrated here that obesity triggers differential regulation of various components of the HSR in obese non-diabetic subjects and a 3-month physical exercise was sufficient to restore the normal expression of HSPs in the adipose tissue with concomitant attenuation in the inflammatory response. This suggests that the in non-diabetic obese human the body is still able to cope with the obesity-related cellular stress via an upregulation of key cytoprotective HSPs in adipose tissue and PBMCs. Given the well documented decrease of HSPs in insulin-resistant and diabetic subjects, it might be interesting to enhance those proteins in non-diabetic obese and sustain their over-expression to prevent the development of further metabolic disorders including diabetes. Further studies are warranted to examine the expression levels of HSPs in visceral adipose tissue and other organs of non-diabetic obese before suggesting precise biological significance of increased HSPs expression due to obesity and the beneficial effects of physical exercise.

## Competing interests

The authors declare that they have no competing interests.

## Authors’ contributions

AT and KA contributed to the study design, performing experiments, analyzing data, writing and reviewing the manuscript. JA contributed to performing experiments, analyzing data, and reviewing the manuscript. MA contributed to manuscript reviewing. IA, PC, JJ, SK, SW contributed to the experiments and data analysis. NE and FA contributed to the study design and data analysis. SD contributed to the study design and manuscript reviewing. MD contributed to the study design, analyzing data, writing and reviewing the manuscript. All authors read and approved the final manuscript.

## Supplementary Material

Additional file 1: Table S1 Primer sequences used for real time PCR to analyze gene expression status of heat shock-related genes. Click here for file
