# Supplementary material for: Immunohistochemical profiling of the heat shock response in obese non-diabetic subjects revealed impaired expression of heat shock proteins in the adipose tissue
Source: Lipids Health Dis. 2014 Jul 1;13:106. doi: 10.1186/1476-511X-13-106 (PMC4085713; doi:10.1186/1476-511X-13-106)
Supplement: Additional file 1: Table S1 — Primer sequences used for real time PCR to analyze gene expression status of heat shock-related genes. [file 1476-511X-13-106-S1.doc]

**Table S1:** Primer sequences used for real time PCR to analyze gene expression status of heat shock-related genes

| **Genes** | **Forward primers** | **Reverse primers** |
| --- | --- | --- |
| *dnajb3* | 5’-ATCCGAGGCCATCAAGAAG-3’ | 5’-CCACCTGCTTGAATCTCCTC-3’ |
| *Hsp-60* | 5’-GATGTCCTGGGCTGTTTCAT-3’ | 5’-GCCTCGATCAAACTTCATGC-3’ |
| *hsp70* | 5’-CGACCTGAACAAGAGCATCA-3’ | 5’-AAGATCTGCGTCTGCTTGGT-3’ |
| *hsp72* | 5’-GGGAGCTGAGTTGCTGGTAG-3’ | 5’-ACCTTGCCATGTTGGAAGAC-3’ |
| *Hsp-90* | 5’-ACTTAGCCAAGATGCCTGAGG-3’ | 5’-CACCCCCAAGAAGTTCACAC-3’ |
| *gapdh* | 5’-AGGGCTGCTTTTAACTCTGGT-3’ | 5’-CCCCACTTGATTTTGGAGGGA-3’ |
